# Biomechanical cell regulatory networks as complex adaptive systems in relation to cancer

**DOI:** 10.1186/s12935-017-0385-y

**Published:** 2017-02-01

**Authors:** Liviu Feller, Razia Abdool Gafaar Khammissa, Johan Lemmer

**Affiliations:** Department of Periodontology and Oral Medicine, Sefako Makgatho Health Sciences University, Pretoria, 0204 South Africa

**Keywords:** Mechanotransduction, Tensional homeostasis, Complex adaptive system, Cancer

## Abstract

Physiological structure and function of cells are maintained by ongoing complex dynamic adaptive processes in the intracellular molecular pathways controlling the overall profile of gene expression, and by genes in cellular gene regulatory circuits. Cytogenetic mutations and non-genetic factors such as chronic inflammation or repetitive trauma, intrinsic mechanical stresses within extracellular matrix may induce redirection of gene regulatory circuits with abnormal reactivation of embryonic developmental programmes which can now drive cell transformation and cancer initiation, and later cancer progression and metastasis. Some of the non-genetic factors that may also favour cancerization are dysregulation in epithelial-mesenchymal interactions, in cell-to-cell communication, in extracellular matrix turnover, in extracellular matrix-to-cell interactions and in mechanotransduction pathways. Persistent increase in extracellular matrix stiffness, for whatever reason, has been shown to play an important role in cell transformation, and later in cancer cell invasion. In this article we review certain cell regulatory networks driving carcinogenesis, focussing on the role of mechanical stresses modulating structure and function of cells and their extracellular matrices.

## Background

Oral squamous cell carcinoma is a malignancy of oral epithelial cells (keratinocytes) characterized by uncontrolled cell proliferation, increased cell survival and disruption of local tissue structure, with malignant keratinocytes invading the underlying connective tissue through the basement membrane. The acquisition of a malignant phenotype is accompanied by several changes in cellular properties including alterations in cell shape, rearrangement of the cytoskeleton, dysregulation in cell-to-cell communication and adherence, and loss of tensional homeostasis. These changes in the function and structure of cells are caused by major reprogramming of gene-expression profiles, by genetic mutations and by non-genetic microevironmental factors including chronic inflammation and trauma, and by altered mechanical properties of extracellular matrix (ECM) [1–3].

The biomechanical characteristics of cancer cells and their immediate microenvironment, and the physical interactions between these cells and their extracellular matrices play important roles in cancer initiation and progression [1, 4, 5]. The ECM of the cancer cells is stiffer than normal owing to increased production of collagen type 1 by tumour associated fibroblasts and to lysyl-oxidase mediated cross-linking of collagen fibers [6–8]. In turn, in response to stresses within the ECM there will be isometric tension in the cancer cells with alterations in the cytoskeletal structural architecture [9, 10]. This results in abnormal activation of certain transcription factors that regulate the expression of genes involved in cell attachment, proliferation, differentiation, migration and apoptosis, thus promoting cancer progression [1, 6, 8–10].

Reciprocally, tension and contractile forces generated by the intracellular actin filaments of the cytoskeleton in response to external biomechanical stimulation are transferred to the ECM, with abnormal remodelling of its three dimensional organization [10]. In the context of cancer, the altered ECM can dysregulate integrin expression and activation, the assembly of focal adhesion proteins, cytoskeletal structure and cell-to-cell and cell-to-ECM adhesions, thus disturbing tensional homeostasis, further promoting cancerization [5, 7, 8, 10, 11].

In this article we review certain cell regulatory networks driving carcinogenesis, focusing on the role of mechanical stresses that modulate normal function and structure of cells and their ECM, and promote carcinogenesis.

## Complex adaptive systems in the context of gene regulatory network

The dynamic cross-talk between numerous genes, and their organization within the gene regulatory network will determine cell structure and function [2]. In turn, normal structure and function of cells are maintained by ongoing complex adaptive processes in the intra-cellular molecular pathways, controlling the overall profile of gene expression. The function of each intracellular molecular pathway is influenced by other such pathways, and the aggregate of the integrated activity of the several interacting pathways determines the biological properties of the cell. The aggregate of this integrated activity is not linear and cannot be derived from summation of the activity of the singular pathways [9, 12, 13].

The intracellular molecular pathways form a network, and each pathway in the network may be activated by one or more biological agents in the microenvironment [2, 9]. This complex dynamic adaptive system that maintains the normal biological properties of the cell is robust and can self-reorganize in response to external stimuli, so that the cells adapt to microenvironmental changes without losing functional integrity [2, 9, 12, 13]. The adaptive system uses a built-in ‘set of rules’ that directs the behaviour of the system almost predictably [12, 13].

The larger system of the gene regulatory network comprises many regulatory circuits, each circuit having a particular profile of gene expression. The configuration of the specific circuit and interactions between the genes within the circuit determine whether a regulatory circuit remains stable, or is driven to adapt to one of multiple accessible stable attractor states in the network, each of which has a distinct profile of gene expression and is associated with an orderly biological behaviour [2, 9].

Within the genetic landscape of multiple stable attractor states, a cell can switch from one stable attractor state to another in response to stimuli from the microenvironment, for example from a proliferative to a differentiative programme. Furthermore, mutations in certain key regulatory genes can induce changes in the profile of the gene network, resulting in cell transformation and cancer development [2, 9]. However, if a mutation is insufficient to cause cell transformation, the attractor state can adapt through self-reorganization, preserving the normal functional integrity of the cell [2].

Thus, the multiple discrete stable attractor states represent pre-existing circuits of gene expression each associated with a different preprogrammed biological behaviour, and according to the built-in rules the switch to a particular stable attractor state will depend on the accessibility of potential target attractors within the gene landscape [2].

The regulatory network that controls tensional homeostasis probably comprises several different attractor states, depending upon the structural architecture of the cells and their ECM. While a normal tensional attractor state maintains the physiological structure of cells and tissues, stiffer matrices with altered tensional homeostasis can switch from a physiological tensional circuit to an aberrant tensional circuit promoting a cellular change to a malignant phenotype [7, 11]. Such a process may be driven by extraneous forces inducing biomechanical signals in the microenvironment such as repetitive trauma, with consequent alteration in the intracellular tension, or by alterations in genes encoding proteins involved in maintaining tissue tensional homeostasis.

## Circuits of gene expression in the context of cancerization

Preset circuits of gene expression (stable attractor states) include some developmental programs that are normally shut down post-developmentally; but random genetic mutations may induce rewiring of gene-regulatory systems, making developmental circuits re-accessible and re-activated, thus providing ‘cancer stem cells’ with renewed capacity to initiate cancer development. Chronic inflammation or trauma as well as other stimuli from the microenvironment might also promote the transition to a preset circuit of gene expression associated with cancer [2]. The cancer stem cells resulting from this process give rise to transient-amplifying cancer cells which can proliferate, degrade extracellular matrix, invade, migrate and promote neoangiogenesis [2].

Acquisition of a malignant phenotype is accompanied by changes in cellular structure and function caused by reorganization of the cytogenetic regulatory network with the re-programming of gene-expression profiles [2]. Thus, such re-organization may be induced by sequential, random gene mutations [14], by natural-selective pressures in the local microenvironment [14], or by biomechanical and biochemical signals originating in the stroma of the cancer [2], all influenced by dysregulated reciprocal interactions between the affected epithelium and the underlying lamina propia [14, 15].

## Biomechanics of the cytoskeleton, focal adhesions and extracellular matrix

**Fig. 1:**
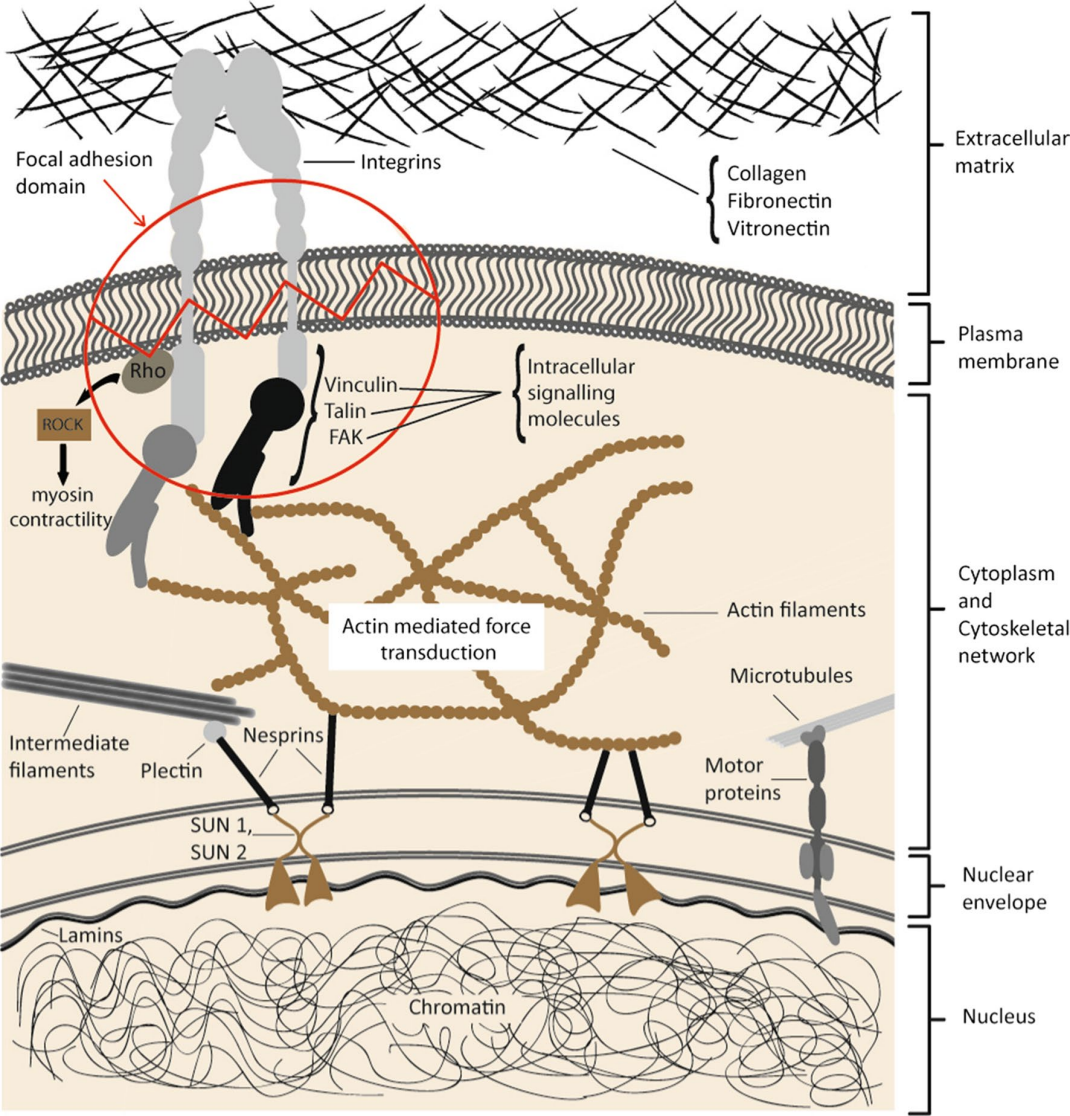
The physical connection between the cell’s plasma membrane and the nuclear envelope via the actin cytoskeleton network, enables transduction of extra- and intracellular mechanical stimuli to reach the nucleus and to activate transcription factors which determine gene expression and subsequent cellular responses. Strains derived from extracellular matrix induce configurational changes in the focal adhesion protein talin, resulting in the recruitment of vinculin with the establishment of a physical link between the ECM and the nuclear membrane via the integrin/talin/vinculin—cytoskeleton—nuclear envelope proteins (Sun 1, Sun 2, Nesprins). Thus extracellular stresses, stiffness of ECM and the mechanical properties of the intracellular actin cytoskeleton influence cell shape and orientation and play a role in the regulation of cell differentiation, proliferation and survival [18, 19, 23]. The figure is adapted from Jaalouk and Lammerding [18] and Feller et al. [19]

The cytoskeleton is a complex of elements comprising actin filaments and intermediate filaments under tension, and of microtubular elements that resist compression, together maintaining the shape and mechanical stability of the cell and its nucleus. These interconnecting cytoskeletal elements are anchored to focal adhesion proteins and integrins on the inner side of the cell’s plasma membrane, and to other specialized proteins on the outer side of the nuclear membrane. Nesprins, the outer nuclear membrane proteins connect the nucleus to the cytoplasmic cytoskeleton, and interact with inner nuclear proteins sun as sun 1, sun 2 and laminins (Fig. 1) [16–19]. In response to physiological biomechanical cues from the ECM, the cytoskeletal elements can self-rearrange, changing their structural configuration to preserve the shape and mechanical stability of the cell [3, 16, 20, 21].

The structural architecture of the cytoskeleton determines its mechanical properties, and in general, the cytoskeleton remodels and stiffens in response to externally- or internally-generated stresses, providing increased resistance to deformation. At the same time, the biomechanical signals from the ECM, transduced via the cytoskeletal structural network, mediate cell proliferation, differentiation, survival and motility, thus ultimately influencing cellular responses [3, 9, 19, 21–23]. In addition, cross-linking between cytoskeletal actin and myosin filaments occurs in response to extracellular mechanical stresses, and the consequently generated intracellular contractile forces activate intracellular signalling pathways, regulating gene expression. These intracellularly generated forces can also be transmitted to the ECM, which consequently may become stiffer [19, 21, 24, 25].

The physical microenvironment of all cells is a complex mesh-like three-dimensional scaffold within the ECM. The biochemical composition, the fibrous architecture and the mechanical properties are important factors determining cell anchorage, proliferation or differentiation, survival, and vectors of cell migration. [5, 19, 21, 26, 27].

A cell adheres to and communicates with its ECM by means of focal adhesion domains which are localized multifunctional dynamic protein complexes integrated in its cellular plasma membrane. These complexes comprise transmembrane integrins, and cytoplasmic proteins including focal adhesion kinase (FAC), talin, vinculin, Src, paxillin and tensin, linking the ECM to the cytoskeleton (Fig. 1). The extracellular component of the integrin interacts with ECM ligands including fibronectin, vitronectin and collagen, and the intracellular component of the integrin interacts with the actin cytoskeleton and other proteins of the focal adhesion domain [3, 18, 21, 27, 28]. The elements of the focal adhesion domain act as cellular mechanosensors and transduces, conveying biomechanical signals from mechanical stresses in the ECM into the intracellular microenvironment, eliciting biochemical signals that induce cellular responses [19, 22, 29, 30].

The focal adhesion domains thus mediate interactions between cells and their matrices, with ECM stresses promoting integrin clustering, expression and activity, and assembly and maturation of focal adhesion proteins with their consequent stabilization [19, 22, 30]. A softer more flexible matrix cannot transmit the stresses necessary for the development and stability of mature cell-to-ECM adhesions [4, 19, 27, 31]. On the other hand, a stiff ECM transmits the strains necessary to bring about and to maintain stronger cell-to-ECM focal adhesions.

The strains that develop in integrins and in other mechanosensory molecules in the focal adhesion domains are generated by both intracellular and extracellular stresses [18, 19]. Extracellular mechanical stresses, through the stimulation of integrins and other focal adhesion molecules, can activate the intracellular Rho-associated kinase (ROCK) pathway, triggering the assembly of bundles of contractile actomyosin called stress fibers that are attached intracellularly at focal adhesion sites [19, 32]. The small G-protein Rho is a member of the Rho family of small guanosine triphosphatases (GTPase), and together with ROCK, they upregulate the phosphorylation of myosin light-chains [21, 26, 28, 33]. Actin polymerization with subsequent cross-linking between the cytoskeletal actin and myosin filaments will give rise to stress fibers, increasing the cytoskeletal tension and contractility [19, 34].

Contraction of the intracellular Rho-ROCK-induced stress fibers further promotes clustering and strengthening of integrins and other molecules in the focal adhesion domain [8]. Contraction of the stress fibers can also activate the mitogen-activated protein kinase-extracellular signal-regulated kinase (MAPK-ERK) signalling pathway that mediates the activity of transcription factors regulating expression of genes involved in cell proliferation and differentiation [10, 16, 22, 28].

Alteration in the biochemical composition and structural architecture of the ECM together with an aberrant increase in its stiffness can cause dysregulation in the intracellular contractile forces which may disrupt cell-to-cell and cell-to-ECM adhesion [35].

Under physiological conditions, a positive biomechanical feedback loop is created, with ECM stresses inducing intracellular stresses, mediating cellular function and biological behaviour; and reciprocally, the intracellular stresses inherent in the cytoskeleton and stress fibers influence remodelling, function and biomechanical properties of the ECM [19].

The functional activities of the cells are thus influenced by the complex adaptive network that regulates the ECM and cellular mechanotransduction pathways. The elements of this complex system include the structural architectures of both the ECM and the cytoskeleton, which impart the inherent mechanical tension and stiffness characteristic to the cells and their matrices. The arrangement of these elements is dynamic, with particular intracellular molecular signalling pathways being activated according to the biomechanical stimuli received from the microenvironment, and as the stimuli change, so do the intracellular signalling pathways, in order to elicit a homeostatic biological response [9, 22].

## Biomechanics and cancer

Development of cancer is associated with increased stiffness of the local ECM that can then transmit a greater portion of any intrinsically generated or extraneous mechanical stress to the cells, transducing the microenvironmental biomechanical stimuli into intracellular biochemical signals. These signals increase intracellular mechanical stresses which then modulates cellular genes expression, promoting cell proliferation, survival and migration [1, 4, 7, 19, 22, 31, 33]. Any increase in the stiffness of the extracellular microenvironment is also associated with an increase in protein concentration in the ECM and also with increased cross-linking and reorientation of the collagen fibrils [6–8, 29, 31].

Low-grade repetitive injury is a local factor that can induce a long-term increase in the stiffness of the ECM, and in the dynamics of cell-to-cell and cell-to-matrix adhesion and of cytoskeletal organization with a consequent increase in cell stiffness, proliferation, survival and migration further promoting cancerous transformation of cells [1, 11, 19, 22, 36].

Transformed cells have an altered profile of intermediate filaments, of cytoskeletal structure and of cell shape compared to normal cells [7] and the abnormally high mechanical stresses and contractile forces generated within and around cells in response to biomechanical stimulation disrupt cell-to-cell junctions and cell-to-matrix adhesions thus facilitating cell migration and invasion [7, 11]. In advanced cancers, the rapidly growing tumour mass generates internal compressive stresses that cause further mechanical alterations in the microenvironment, further modification of the ECM proteins, and greater cellular transformational responses [7]. The abnormally stiff ECM in advanced cancers then induces abnormal Rho-ROCK pathway-mediated cellular contractility with increased isometric tension, and with chronic activation of the MAPK-ERK (proto) oncogenic pathway, all of which are necessary to maintain an invasive phenotype of cancer cells [6, 8, 11].

The transmitted mechanical stresses, via the changed and stiffer cytoskeletal structure, can be transmitted to the nucleus causing nuclear deformation with configurational changes in chromatin, thus influencing gene expression which independently of the biomechanical-induced biochemical changes have the potential to promote cancerization (Fig. 1) [7, 19].

The mechanical properties of cancer cells change during cancer progression, with metastatic cells having left the site of the primary tumour to migrate through a different type of extracellular microenvironment, becoming less stiff than they were after initial transformation, to allow penetration of the basement membrane and underlying tissue [1, 29, 37, 38].

In the context of cellular circuits of gene expression and cancerization, it has become evident that extracellular mechanical stresses generated by an aberrant increase in ECM stiffness has the capacity to induce rewiring of the epithelial mesenchymal transition (EMT) developmental cellular circuit [39, 40]. EMT transcription factors including Twist 1, Twist 2, Snail, Slug, ZEB 1 and ZEB 2 can in epithelial cells suppress E-cadherin expression resulting in functional loss of cell-to-cell adhesion and induce alterations in the actin cytoskeleton, which alterations can mediate the conversion of polarized immobile epithelial cells to mobile cells with a mesenchymal phenotype [19, 23, 41].

In addition to stiff matrices and other mechanical stresses, TGF-β, Notch, Wnt and Hedgehog signalling pathways can also activate EMT transcription factors [19, 42]. With regard to mechanical stresses, it has been shown that high-matrix stiffness-driven Twist 1 mechanotransduction signalling pathways interact with TGF-β signalling pathways to promote tumour invasion and metastasis [39, 40].

It appears that there are different modes of cancer cell migration that are governed by different genetic programs, based on the specific characteristics of the cell-to-cell junctions, cell-to-ECM adhesions, the tension of the cytoskeleton and the patterns of remodelling of all these elements. The modes of cell migration change over-time in response to microenvironmental biomechanical cues [43].

## Conclusion

Cancer initiation and progression are associated with pathological changes in structure and function of cells and of their ECM, and with disruption of tensional homeostasis characterized by abnormal mechanical stresses within cells and their extracellular matrix. The aberrant process of mechanotransduction which converts biomechanical stimuli into abnormal intracellular biochemical signals is thought to play an important role in early cellular transformation.

## Abbreviations

ECM: extracellular matrix
FAC: focal adhesion kinase
GTPase: guanosine triphosphatases
ROCK: Rho-associated kinase
MAPK-ERK: mitogen-activated protein kinase-extracellular signal-regulated kinase
